# The Unrelenting Non-sporulating Hyphae

**DOI:** 10.7759/cureus.15176

**Published:** 2021-05-22

**Authors:** Sharan Silvarajoo, Chan Jan Bond, Embong Zunaina

**Affiliations:** 1 Ophthalmology, Hospital Kuala Lumpur, Kuala Lumpur, MYS; 2 Ophthalmology, International Specialist Eye Centre, Kuala Lumpur, MYS; 3 Ophthalmology, Department of Ophthalmology and Visual Science, School of Medical Sciences, Health Campus, Universiti Sains Malaysia, Kota Bharu, MYS

**Keywords:** non-sporulating hyphae, fungal endogenous endophthalmitis, molds, aspergillus endophthalmitis, diabetes mellitus

## Abstract

A 42-year-old man presented with generalized redness in the left eye and painless blurring of vision for four days. He also had a fever and a large left leg abscess for four days prior to the onset of eye symptoms. Visual acuity of the left eye was hand movement with a positive relative afferent pupillary defect. Conjunctiva was injected with chemosis and mild corneal haziness centrally. There was a presence of whitish fibrin covering the pupil and presence of hypopyon with anterior chamber inflammatory cells. The fundoscopic view was obscured by the presence of fibrin in the pupillary area. B-scan ultrasound showed severe vitritis with multiple loculations. He was treated as left eye endogenous endophthalmitis secondary to left leg abscess. He was given multiple intravitreal antibiotic injections together with intravenous ceftazidime and gutt. moxifloxacin. The vitreous specimen did not yield any growth. Incision and drainage were done for the left leg abscess, and yellowish pus was aspirated with negative culture. Trans-pars plana vitrectomy was performed in view of poor clinical response. However, despite that, his left visual acuity dropped to non-perception of light (NPL). The vitreous specimen taken during vitrectomy finally showed non-sporulating fungal hyphae. He was started on oral fluconazole and topical amphotericin B. His left eye remains as NPL. However, his general eye condition improved.

## Introduction

Endogenous fungal endophthalmitis is an alarming ocular emergency associated with poor visual outcomes, as it can cause blindness [1-2]. Most patients with this diagnosis have predisposing systemic risk factors such as diabetes mellitus [3-4]. It rarely occurs in healthy immunocompetent individuals. The most common organism causing endogenous fungal endophthalmitis are the yeast species *Candida albicans*, followed by *Aspergillus* species [5-6]. We report a case of endogenous fungal endophthalmitis in an immunocompromised patient that has caused blindness. To our knowledge, this is the first reported case of endogenous fungal endophthalmitis caused by non-sporulating hyphae in Malaysia.

## Case presentation

A 42-year-old man, a known case of type 2 diabetes mellitus, presented with a generalized painless blurring of vision in the left eye for four days. It was associated with left periorbital swelling and eye redness. He had a fever with a left leg abscess for four days prior to eye symptoms. He was treated with an oral antibiotic for the leg abscess.

On examination, he was not toxic-looking and afebrile. His vital sign was stable with a blood pressure of 120/84 mmHg and the pulse rate was 84 beat per minute. Visual acuity of the right eye was 6/9 and the left eye was hand movement. There was a positive relative afferent pupillary defect on the left eye.

**Figure 1:**
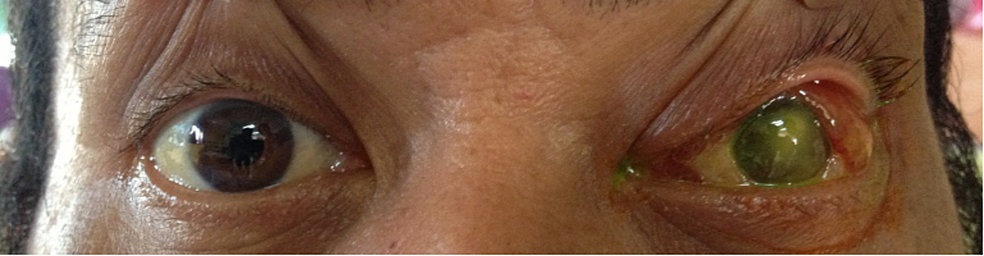
Left eye showed periorbital swelling with generalized redness

**Figure 2:**
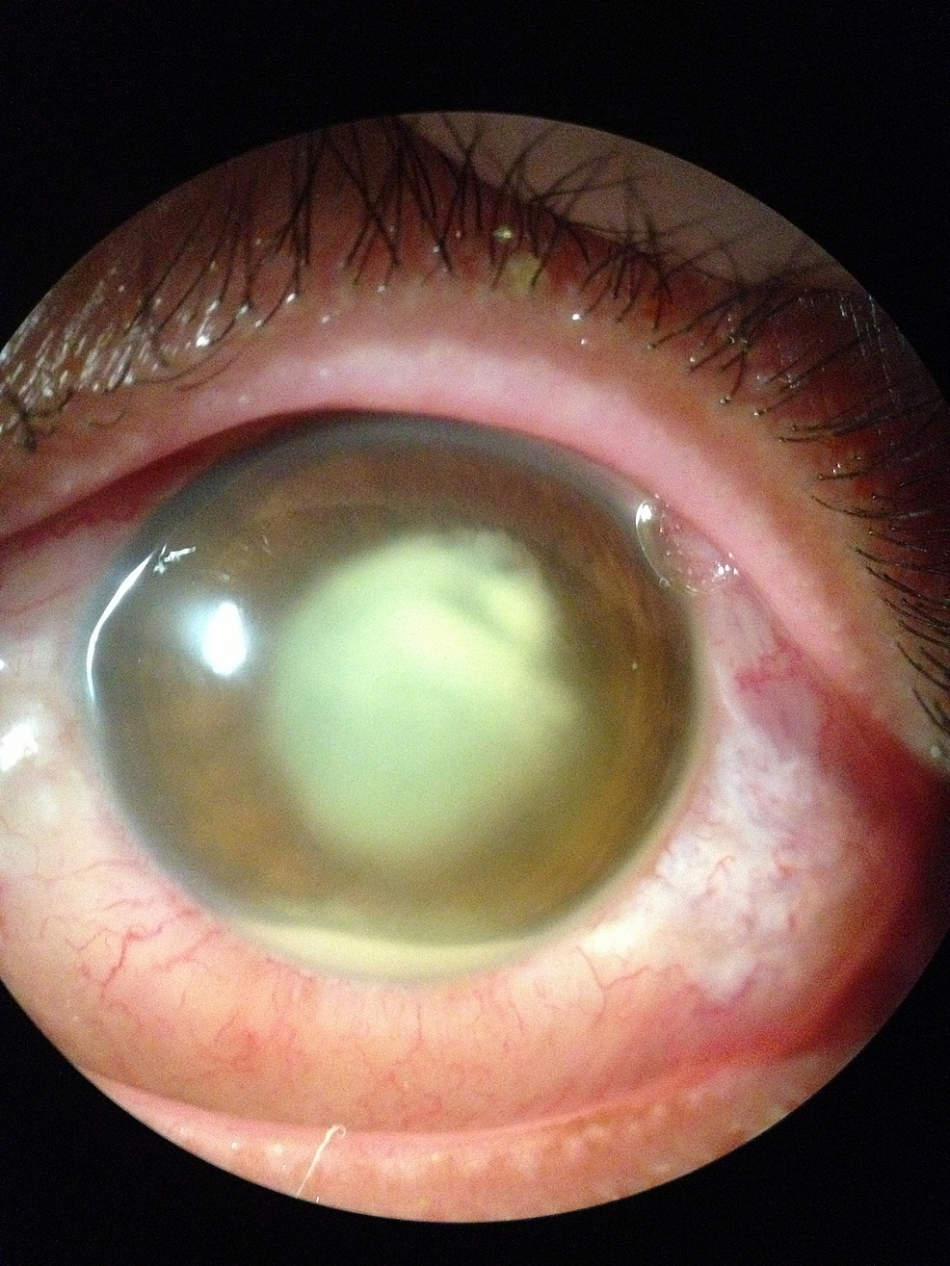
Whitish fibrin covering the pupil with hypopyon

Left eye examination showed periorbital swelling with generalized redness (Figure 1). The conjunctiva was injected with chemosis at the lateral part and mild corneal haziness centrally. There was a presence of whitish fibrin covering the pupil and the height of the hypopyon was 1.5 mm with cells 4+ in the anterior chamber (Figure 2). The pupil was round, 3 mm in size, and reactive to light. Intraocular pressure was 17 mmHg. There was a mild restriction of eye movement in all directions. The fundus view was obscured by the presence of fibrin in the pupillary area. B-scan ultrasound showed severe vitritis with multiple loculations. Right eye examination was normal, with no features of diabetic retinopathy.

**Figure 3:**
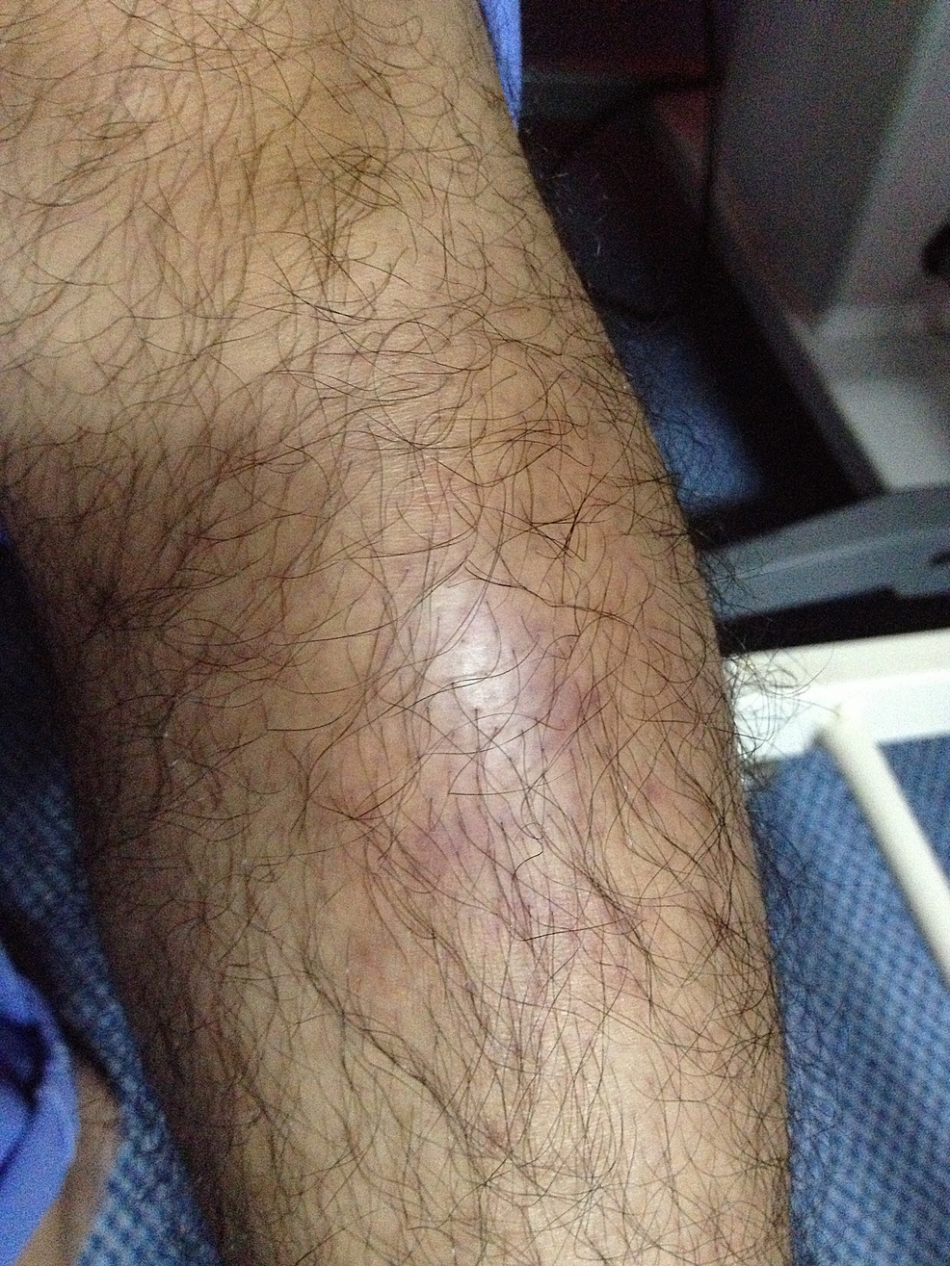
Left leg abscess at the anterior shin

Left leg examination showed a large abscess at the anterior shin, measuring 8 cm x 6 cm (Figure 3). The area of the abscess was red in color, warm, painful on palpitation, with mild to moderate fluctuation. There was no punctum or open sore over the abscess area. There was no other abscess on other parts of the body. The lung was clear, with a normal cardiovascular system. There was no hepatosplenomegaly and no lymphadenopathy.

Capillary blood sugar was 12.0 mmol/L at presentation, and the total white blood cell count was 18.9 x 109/L with predominantly neutrophils (80%). Urine full examination microscopy examination (UFEME) was normal with no growth. Chest X-ray and ultrasound abdomen showed normal findings.

He was treated as left eye endogenous endophthalmitis secondary to left leg abscess. Intravitreal vancomycin 2 mg/0.1 ml with ceftazidime 2 mg/0.1 ml injections were given to the left eye. Vitreous specimens for culture and sensitivity did not yield any growth. He was also started with intravenous ceftazidime 1 gm TDS and gutt. moxifloxacin hourly to the left eye. Incision and drainage were done for the left leg abscess. About 15 ml of yellowish pus was drained during the procedure. Pus and tissue culture did not grow any organism.

**Figure 4:**
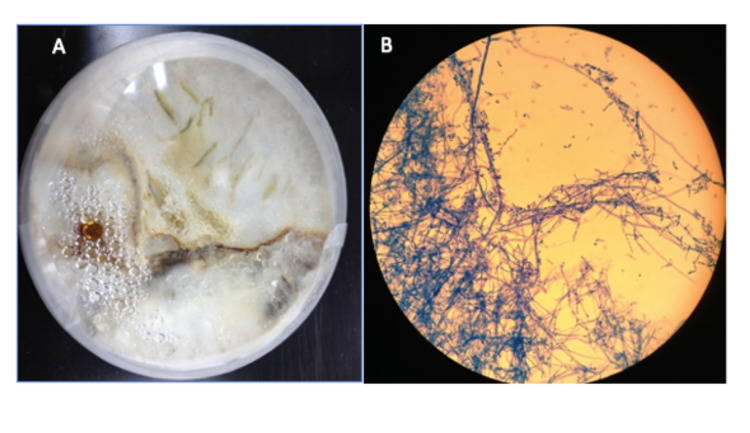
Non-sporulating fungal hyphae from the vitreous specimen on a culture plate (A) and microscopic examination (B)

**Figure 5:**
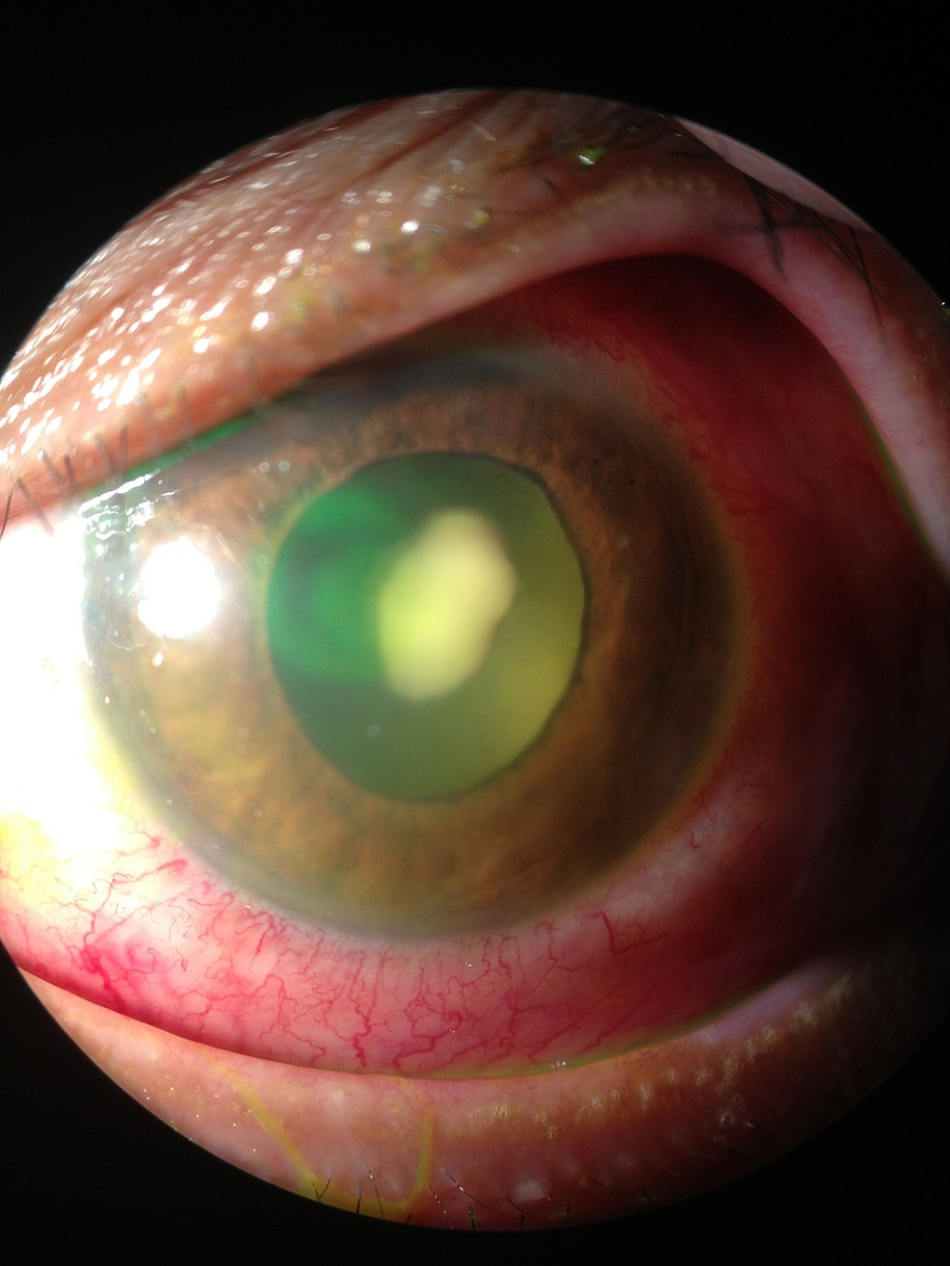
Improvement of eye condition - one-week post vitrectomy

He was given multiple intravitreal antibiotic injections but there was no improvement. In view of the poor clinical response, trans pars plana vitrectomy was performed. Despite that, his left visual acuity dropped to non-perception of light (NPL). His vitreous sample taken during vitrectomy finally showed non-sporulating fungal hyphae (Figure 4). His general eye condition improved (Figure 5) after administration of oral fluconazole 200 mg BD and topical amphotericin B hourly to the left eye. However, his left eye remains NPL. There was a resolution of periorbital swelling and chemosis with full extraocular muscle movement. The hypopyon was resolved with contracted fibrin and mild anterior chamber reaction. The oral fluconazole was continued to be completed for six weeks and the topical amphotericin B was tapered down slowly within three months.

## Discussion

Endophthalmitis is a devastating eye condition, as it can cause irreversible blindness. The word “endophthalmitis” refers to inflammation of the intraocular fluids (vitreous and aqueous) usually due to infection. Endophthalmitis can be either exogenous or endogenous. An exogenous infection is commonly seen after cataract surgery, post eye trauma, bleb-related, or due to cornea ulcer, whereas an endogenous infection originates from hematogenous seeding of pathogens during bacteremia or fungemia [1].

Endogenous endophthalmitis is reported as 7.7%-13.2% [1] amongst endophthalmitis. It is a rare complication of sepsis, found in less than 0.5% of patients with fungemia and 0.04% of patients with bacteremia [2]. The risk factors include diabetes mellitus, human immunodeficiency virus, intravenous drug abuse, renal failure on dialysis, cardiac disease, malignancy, immunosuppressive therapy, or indwelling catheters [3].

Fungal endogenous endophthalmitis is commonly caused by yeast (eg. *Candida*) or molds (eg. *Aspergillus, mycelia*). Patients with endogenous endophthalmitis caused by molds (non-sporulating fungi) have a fulminant course of disease manifest by a shorter duration of symptoms, worst presenting vision with hypopyon and virulent in nature, with poor visual outcomes requiring trans pars plana vitrectomy; and some require evisceration [3]. This is in keeping with our patient who presented with visual acuity of hand movement with hypopyon within four days of the leg abscess. In contrast, patients with endogenous endophthalmitis caused by yeast present more indolently with better visual acuity and outcomes [3].

Non-sporulating fungi refer to molds that fail to produce spores either because they have lost this ability, or conditions were not suitable, or required very long periods to produce spores [7]. Examples of these organisms include *Aspergillus*, *mycelia sterilia*, *Cladosporium*, and *Alternaria*. On tape lift samples, these non-sporulating fungal may appear colorless or pigmented (brown), septate (with cross-walls), or non-septate [7-8]. Our patient obtained pigmented brownish branching hyphae in his culture plate corresponding to non-sporulating fungi.

Fungi that do not sporulate in culture do produce spores in nature, can produce allergens and irritants, and can cause systemic infection in immunocompromised patients [2,4]. Identification of the exact non-sporulating hyphae is not possible unless sporulation can be induced, which requires weeks to months. Hence, the diagnosis of fungal endophthalmitis caused by non-sporulating hyphae can be missed due to its features of slow/delay in growing culture. Hence, clinical judgment plays an important role in the diagnosis of non-sporulating fungal endophthalmitis.

Essman et al. have quoted in their literature that prompt diagnosis and aggressive treatment could hasten the recovery and prevent the need for evisceration in fungal endogenous endophthalmitis [5]. This is also agreeable by Binder et al. in his writing on treatment outcomes in fungal endophthalmitis patients [6].

The diagnosis of fungal endogenous endophthalmitis is highly through clinical judgment supported by cultures of vitreous or aqueous. Prompt referral by a multidisciplinary team due to eye symptoms in patients with systemic infection plays an important role in rapid diagnosis and treatment by ophthalmologists. A high index of suspicion should be raised in patients with risk factors such as diabetes mellitus. The prognosis for fungal endophthalmitis is generally poor; however, timely diagnosis and treatment with systemic antifungal followed by vitrectomy could prevent the need for evisceration to save the globe and offer the possibility of better visual outcomes, which can lead to a better quality of life.

## Conclusions

The diagnosis of fungal endophthalmitis should be highly suspected in an immunocompromised patient if the patient has a poor clinical response despite the administration of antibiotics. Early diagnosis with treatment can prevent the need for evisceration, which could be devastating and may lead to poor quality of life.
